# Pathogenic truncating filamin C mutations presenting as acute myocarditis: a case series with insights from cardiac magnetic resonance and histological analysis

**DOI:** 10.1093/ehjcr/ytae111

**Published:** 2024-02-27

**Authors:** Apostolos Vrettos, Polyvios Demetriades, Martín Ortiz, Fernando Domínguez, Pablo García-Pavía, M Paz Suárez-Mier, Thomas Gossios, Konstantinos Savvatis

**Affiliations:** Barts Heart Centre, Barts Health NHS Trust, St Bartholomew’s Hospital, West Smithfield, London, UK; Barts Heart Centre, Barts Health NHS Trust, St Bartholomew’s Hospital, West Smithfield, London, UK; Departamento Científico/Scientific Department, Health in Code, Edificio O Fortín, Hospital Marítimo de Oza, As Xubias s/n, 15006 A Coruña, Spain; Heart Failure and Inherited Cardiac Diseases Unit, Department of Cardiology, Hospital Universitario Puerta de Hierro Majadahonda, IDIPHISA, CIBERCV, Madrid, Spain; Centro Nacional de investigaciones Cardiovasculares (CNIC), Instituto de Salud Carlos III, Madrid, Spain; Heart Failure and Inherited Cardiac Diseases Unit, Department of Cardiology, Hospital Universitario Puerta de Hierro Majadahonda, IDIPHISA, CIBERCV, Madrid, Spain; Centro Nacional de investigaciones Cardiovasculares (CNIC), Instituto de Salud Carlos III, Madrid, Spain; Histopathology Service, National Institute of Toxicology and Forensic Sciences, Madrid, Spain; Laboratory of Cardiomyopathies and Inherited Cardiac Diseases, Aristotle University of Thessaloniki 1st Cardiology Department, AHEPA University Hospital, 546 21 Thessaloniki, Greece; Inherited Cardiomyopathies Unit, St Bartholomew’s Hospital, W Smithfield, London EC1A 7BE, UK; Institute for Cardiovascular Science, University College London, 62 Huntley St, London WC1E 6DD, UK; NIHR University College London Hospitals Biomedical Research Centre, 170 Tottenham Ct Rd, London W1T 7HA, UK; William Harvey Research Institute, Queen Mary University London, John Vane Science Centre, Charterhouse Square, London EC1M 6BQ, UK

**Keywords:** Case series, Filamin C mutation, Cardiomyopathy, Myocarditis

## Abstract

**Background:**

The exact mechanisms underlying the pathogenesis of myocarditis are not always understood, but there is emerging evidence to suggest that genetic factors may play a significant role.

**Case summary:**

Herein, we present six cases in which clinical, biochemical, and cardiovascular magnetic resonance data were consistent with myocarditis, and genetic testing subsequently revealed pathogenic filamin C (FLNC) mutations. Three patients presented with ventricular arrhythmias, two with severe biventricular dysfunction, and two suffered sudden cardiac arrest. Three received an implantable cardioverter defibrillator, and one underwent heart transplantation. Cascade testing was useful in identifying other relatives with FLNC mutation. We also present relevant histology results of myocardial specimens showing the presence of lymphocytic infiltration and inflammation, further supporting the potential association between FLNC mutations and a myocarditis phenotype.

**Discussion:**

Genetic testing of affected individuals for FLNC mutations and cascade screening in the setting of acute myocarditis may be considered in selected clinical context, such as in acute myocarditis accompanied by severe left ventricular systolic dysfunction, biventricular failure, significant ventricular arrhythmias, or right ventricular involvement.

**Learning points**

- Myocarditis, by clinical and histological criteria, is a common presentation in patients with FLNC mutations. Arrhythmic risk and the risk of sudden death appear to be elevated during periods of myocardial inflammation in patients with FLNC mutations.
- This case series also highlights the potential role of genetic testing in patients presenting with acute myocarditis.
- The diagnostic yield of such testing may be higher in distinct phenotypes (e.g. those with severe myocarditis or ventricular arrhythmias).

## Introduction

Truncating mutations of the filamin C (FLNC) gene can result in abnormal translation of FLNC protein, a sarcomeric structural protein found exclusively in striated muscle.^1^ FLNC gene mutations have been associated with skeletal myopathy, as well as dilated and arrhythmogenic left ventricular cardiomyopathy.^2^ Typically, this is characterized by epicardial or ring-like late gadolinium enhancement (LGE) on cardiac magnetic resonance (CMR) and high risk of ventricular arrhythmias and sudden death even in patients with preserved or only mildly reduced ejection fraction (EF).^3^ Here, we describe several cases of truncating FLNC carriers presenting with clinical evidence of myocarditis, as per established conventional clinical, imaging, or histological criteria (Table 1).^4^

**Table 1:** Summary of clinical characteristics and outcomes of cases

	Age	Sex	Mutation	FLNC truncating mutation	C-reactive protein	Troponin (ng/L)	Ventricular arrhythmias	Relevant CMR/imaging data	Histology	Outcome
Patient 1	19	F	p.Arg991*	Nonsense	11	3000	Accelerated idioventricular rhythm/frequent polymorphic VEs	CMR: basal lateral subepicardial LGE, mildly raised T_2_, LV EF = 59%	—	Alive, ICD
Patient 2	53	M	p.Arg991*	Nonsense	47	96	VT	CMR: extensive subepicardial LGE in basal to mid-anterior and lateral walls, increased STIR signal, LV EF 42%	—	Alive, ICD
Patient 3	51	M	p.Pro1031Argfs*47	Frameshift	150	147	NSVT/frequent VEs	CMR: on presentation—dilated LV and RV, extensive epicardial and RV LGE, and LV thrombus and early gadolinium enhancement. LV EF 10% On follow-up—EF 45–50%	—	Alive, ICD
Patient 4	18	M	Ser2077Argfs*50	Frameshift	—	—	Not known	No CMR done. LV EF on echo 27%	Lymphocytic infiltration of the right ventricle, but no fibrosis	Alive, heart transplant
Patient 5	16	M	Ala656Profs*8 and Val868Asp missense PKP2 mutation of uncertain clinical significance	Frameshift	—	—	Cardiac arrest	n/a	Extensive lymphocytic infiltration and fibrosis	RIP
Patient 6	13	M	p.Arg269*	Nonsense	—	—	Cardiac arrest	*CMR in first-degree relative*: patchy, subepicardial LGE in the basal and mid-inferolateral wall and septum	Extensive lymphocytic infiltration and fibrosis	RIP

EF, ejection fraction; ICD, implantable cardioverter defibrillator; LGE, late gadolinium enhancement; LV, left ventricle; NSVT, non-sustained ventricular tachycardia; RIP means death; RV, right ventricle; STIR, short inversion time inversion recovery; VEs, ventricular ectopic beats; VT, ventricular tachycardia.

## Summary figure

**Figure:**
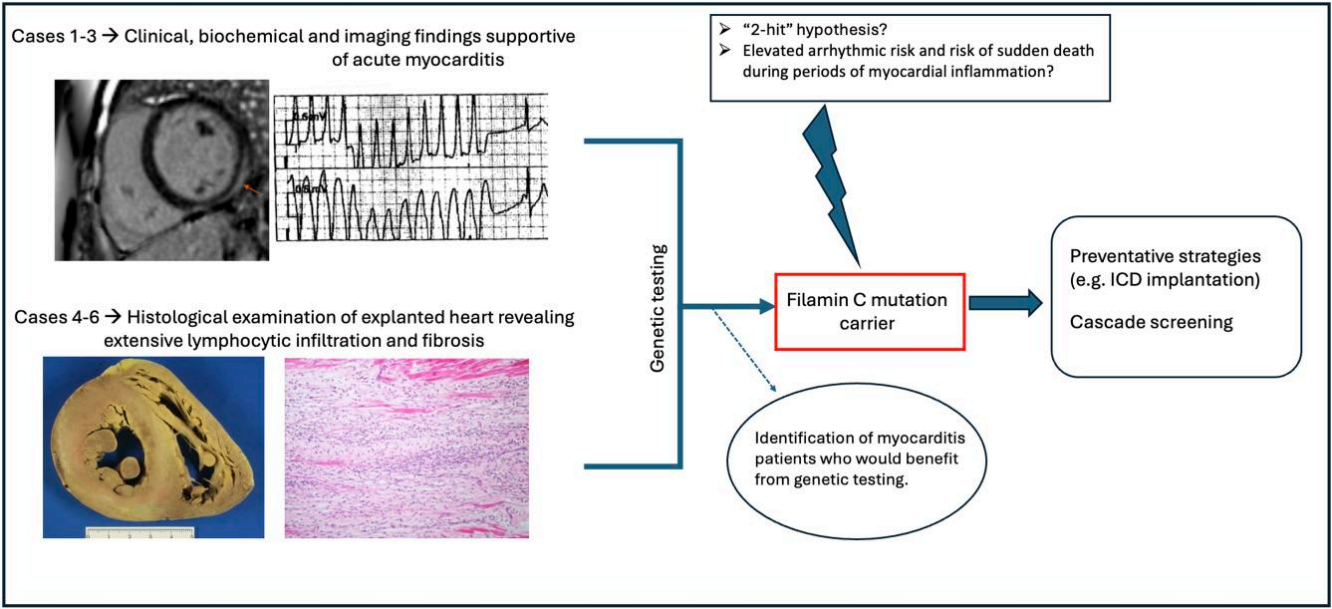


## Patient 1

### Presentation

A 19-year-old female presented to the emergency department (ED) with chest tightness and palpitations following a viral prodrome. Her family history included sudden death of her great-grandmother from maternal side at the age of 59 and of her great-great-grandmother from maternal side at the age of 40.

### Investigations

**Figure 1:**
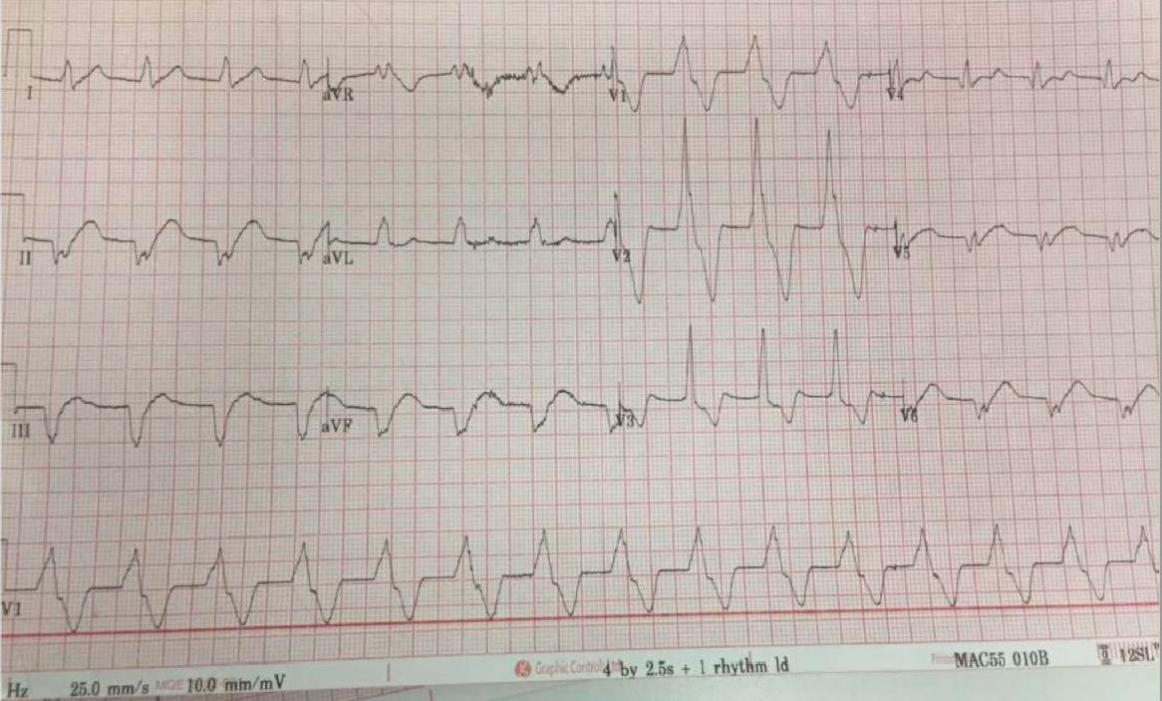
Patient 1. Electrocardiogram on presentation.

**Figure 2:**
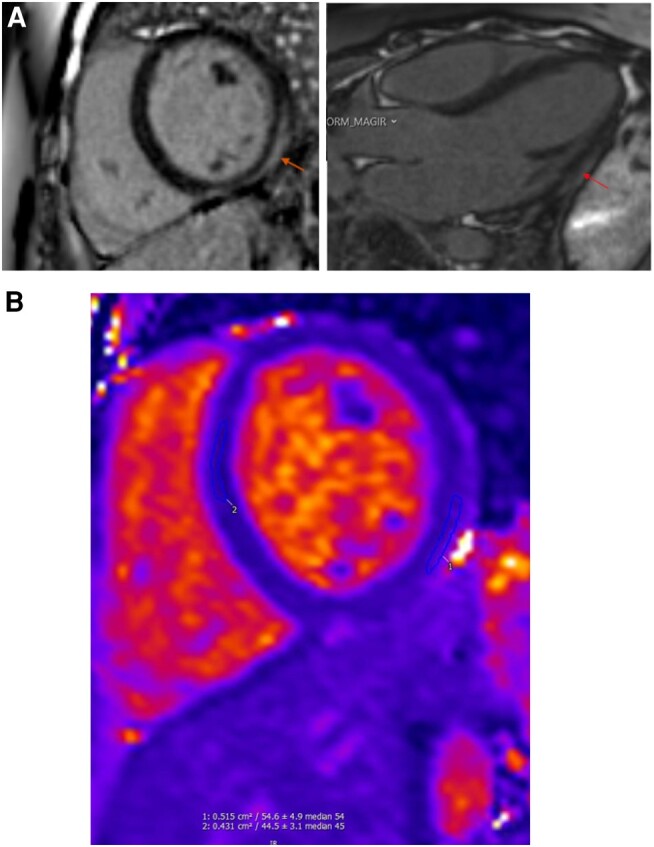
Patient 1. (*A*) Cardiac magnetic resonance scan showing basal lateral subepicardial late gadolinium enhancement (arrow). (*B*) Limited parametric mapping showed mildly increased T_2_ values in the lateral wall (T_2_ = 54 ms, normal range = 40–51 ms at 1.5 T) and normal T_2_ values in the septum (T_2_ = 45 ms).

Her electrocardiogram (ECG) demonstrated an accelerated idioventricular rhythm (*Figure 1*), and her high-sensitivity (hs) troponin T level on admission was markedly elevated at 3000 ng/L (normal range 0–14 ng/L). A CMR scan was performed, which revealed basal lateral subepicardial LGE and a mildly dilated left ventricle (LV) with a preserved EF of 59% (*Figure 2*). These findings were considered to be in keeping with acute myocarditis in the context of significant troponin T elevation. An endomyocardial biopsy was not performed due to the typical clinical presentation and the family history. Genetic testing performed after discharge revealed a pathogenic FLNC p.Arg991* nonsense truncating mutation. Further workup for arrhythmogenic cardiomyopathy (ACM) including signal-averaged electrocardiography, transthoracic echocardiography, and cardiopulmonary exercise testing were normal. A 24-h Holter monitor revealed occasional polymorphic ventricular ectopics.

### Treatment and follow-up

A transvenous implantable cardioverter defibrillator (ICD) was implanted for primary prevention. Further cascade testing led to her mother and maternal grandmother being diagnosed with FLNC mutation with focal scar on CMR and frequent ventricular ectopy on 24-h Holter. On subsequent follow-up visits, the patient has remained asymptomatic and has not received any therapies from her device.

## Patient 2

### Presentation

**Figure 3:**
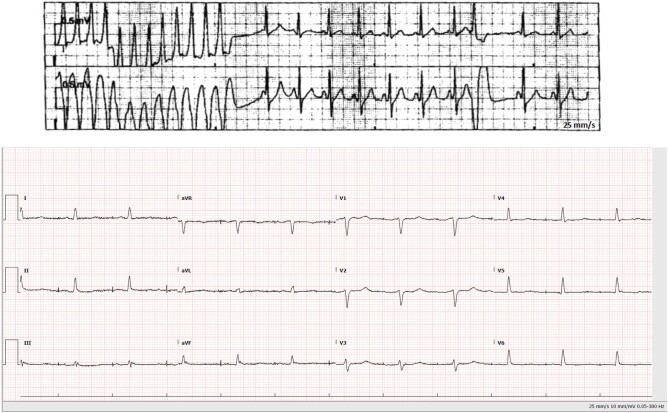
Patient 2. Electrocardiogram showing monomorphic ventricular tachycardia (top trace) and QRS fragmentation in inferior leads (bottom trace).

A 53-year-old male presented to the ED with haemodynamically stable monomorphic ventricular tachycardia (VT; *Figure 3*). He was treated with intravenous (i.v.) amiodarone 300 mg and urgent synchronized direct current (DC) cardioversion, reverting to sinus rhythm. He had a family history of sudden cardiac death of his paternal uncle in his 50s.

### Investigations

**Figure 4:**
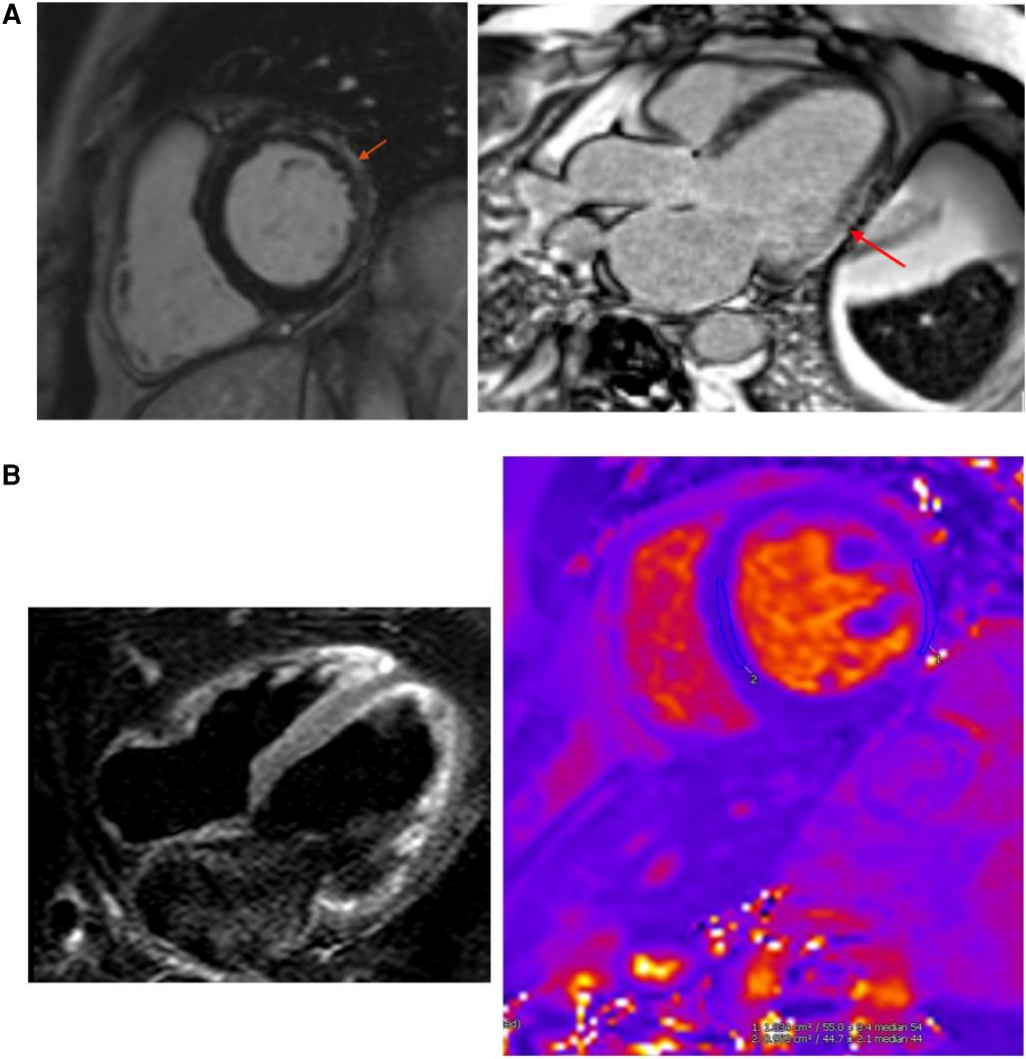
Patient 2. (*A*) Cardiac magnetic resonance scan showing extensive subepicardial late gadolinium enhancement in basal to mid-anterior and lateral walls (arrow). (*B*) Left: increased short inversion time inversion recovery (STIR) signal in lateral wall. Right: also, limited parametric mapping suggested mildly increased T_2_ values in the lateral wall (T_2_ = 54 ms, normal range = 40–51 ms at 1.5 T) and normal T_2_ values in the septum (T_2_ = 44 ms; right).

His resting ECG post DC cardioversion showed QRS fragmentation in inferior leads (*Figure 3*). His troponin on admission was 96 ng/L (normal range 0–14 ng/L) and his C-reactive protein 47 mg/L (normal range 0–5 mg/L). An invasive coronary angiogram was normal. Cardiac magnetic resonance revealed a moderately dilated LV with moderately reduced EF of 42%, with extensive subepicardial LGE in basal to mid-anterior and lateral walls and elevated myocardial native T_2_ values on T_2_ mapping, indicating some degree of myocardial oedema/inflammation (*Figure 4*).

### Treatment and follow-up

The patient was treated with guideline-directed heart failure treatment (initially ramipril 2.5 mg daily, bisoprolol 2.5 mg daily, and spironolactone 25 mg daily, with subsequent up-titration in follow-up appointments), and a transvenous ICD was implanted for secondary prevention. The patient was further investigated as an outpatient with a positron emission tomography (PET) scan and serum angiotensin-converting enzyme, both of which were normal, making a diagnosis of cardiac sarcoidosis unlikely. Genetic testing was then performed, which revealed a pathogenic FLNC p.Arg991* truncating mutation. On subsequent follow-up visits, the patient has remained asymptomatic and has not received any therapies from her device.

## Patient 3

### Presentation

A 51-year-old man presented with acute chest pain, and several-day history of breathlessness and orthopnoea. His past medical history included psoriasis only. He denied smoking, alcohol consumption, or recreational drugs. There was family history of sudden cardiac death in his father at the age of 42, but no other details were available. On examination, he was found to be in cardiogenic shock, requiring inotropic support.

### Investigations

**Figure 5:**
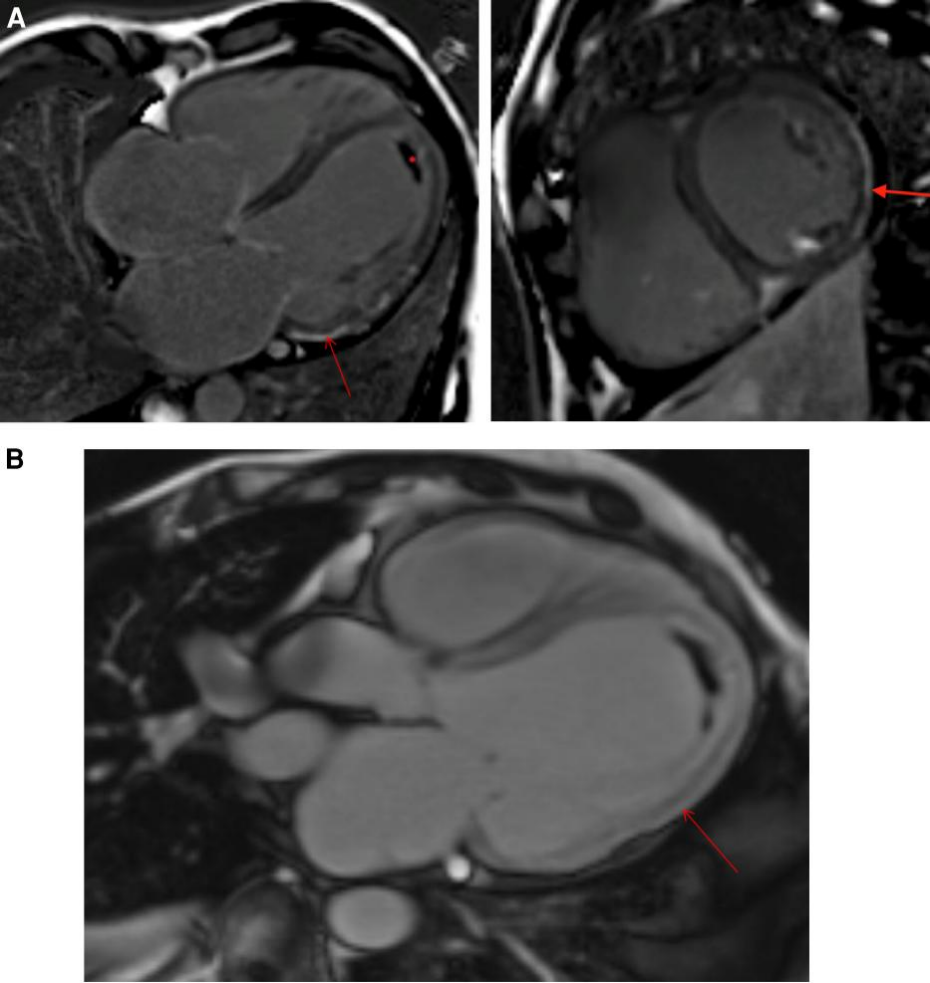
Patient 3. (*A*) Cardiac magnetic resonance scan showing dilated left ventricle and right ventricle, extensive epicardial and right ventricular late gadolinium enhancement (arrow), and left ventricular thrombus (asterisk). (*B*) Early gadolinium enhancement suggestive of hyperaemia and capillary leak (arrow).

His C-reactive protein was 150 mg/L (normal range 0–5 mg/L), his white cell count (WCC) 14 × 10^9^/L (normal range 4.0–11.0 × 10^9^/L), and his troponin 147 ng/L (normal range 0–14 ng/L). His ECG showed sinus tachycardia and his echocardiogram severe biventricular systolic impairment. He had a CMR scan, which showed severely dilated and impaired LV, impaired right ventricle (RV), extensive epicardial LGE, RV LGE, and LV thrombus (*Figure 5*). Coronary computed tomography angiography showed unobstructed coronaries, leading to the clinical diagnosis of myocarditis in view of the CMR findings and troponin elevation.

### Treatment and follow-up

A transvenous ICD was implanted before discharge. He received guideline-directed heart failure therapy (initially ramipril 2.5 mg daily, bisoprolol 2.5 mg daily, and spironolactone 12.5 mg daily, with subsequent up-titration in follow-up appointments), which led to gradual improvement of LV and RV function. Genetic testing revealed FLNC, a pathogenic p.Pro1031Argfs*47 truncating mutation. On his most recent follow-up echo, he had normal biventricular volumes with only mild LV systolic dysfunction with an EF of 45–50% and normal RV function and remains otherwise asymptomatic.

## Patient 4

A 52-year-old male with severe dilated cardiomyopathy (DCM) with symptoms consistent with New York Heart Association (NYHA) class IV was listed for cardiac transplantation. His baseline ECG demonstrated left bundle branch block and sinus rhythm. His baseline transthoracic echocardiogram revealed a left ventricular EF of 27% and a grossly elevated LV diastolic diameter of 83 mm as well as a dilated RV. This patient had undergone genetic testing and was known to have a pathogenic FLNC Ser2077Argfs*50 frameshift truncating mutation. Cascade testing of the patient’s family identified his brother to carry the same mutation, with a phenotype consisting of DCM with palpitations and atrial fibrillation (AF). Histologic investigation of the explanted heart revealed lymphocytic infiltration of the RV with myocardial fibrosis, suggestive of chronic myocarditis.

## Patient 5

**Figure 6:**
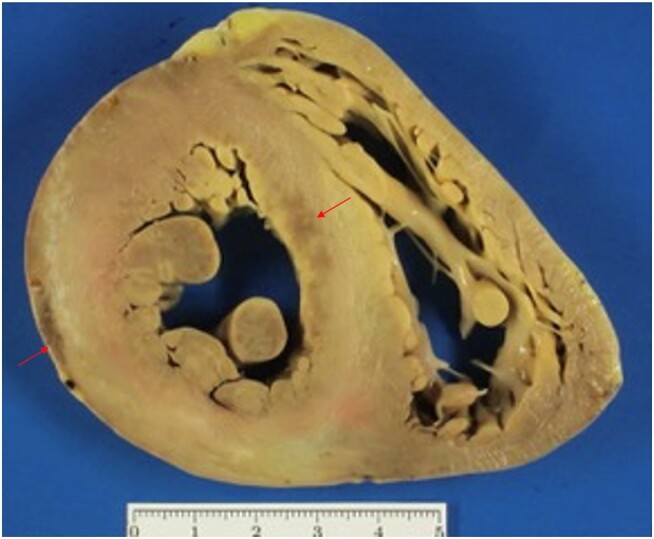
Patient 5. Gross heart axis specimen revealing a darkened septum and left ventricular free wall indicative of extensive myocardial fibrosis (arrows).

**Figure 7:**
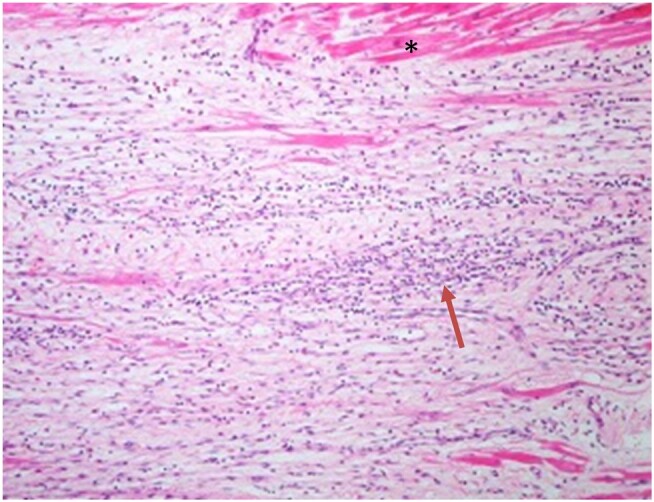
Patient 5. Histological specimen from the septum revealing extensive lymphocytic infiltration (arrow showing lymphocyte-rich inflammatory infiltrate) and fibrosis (the image shows numerous fibroblasts, with only a few cardiomyocytes at the top indicated with an asterisk).

A 16-year-old male suffered a sudden cardiac arrest and had an unsuccessful resuscitation. Post-mortem examination of the heart revealed extensive subepicardial and intramural acute–subacute myocarditis with patches of fibrosis in the LV free wall and septum (*Figures 6* and *7*). No viral genomes were isolated from cardiac tissue. Genetic testing identified the Ala656Profs*8 frameshift pathogenic truncating FLNC mutation as well as a Val868Asp missense PKP2 mutation of uncertain clinical significance.

## Patient 6

A 13-year-old male suffered a sudden cardiac arrest at home. He had developed tonsillitis with systemic symptoms a few days prior to his cardiac arrest. The post-mortem examination of the heart revealed lymphocytic myocarditis. Genetic testing identified a pathogenic, truncating FLNC variant (p.Arg269*). Cascade screening of the first-degree relatives confirmed that the deceased’s brother and mother were also carriers of the same variant. Clinical, ECG, and echocardiographic screening of the proband’s mother did not identify any definitive evidence of structural heart disease, with the exception of mildly increased ventricular ectopic burden on Holter monitoring. Holter monitoring of his brother revealed AF, and his CMR showed focal asymmetry in the interventricular septum and dense, patchy, subepicardial late enhancement in the basal and mid-inferolateral wall and mid-septal segments, and he was referred to the inherited cardiomyopathy service for further management.

## Discussion

FLNC mutations have recently gained interest due to their involvement in the pathogenesis of left-dominant forms of ACM. However, its association with a myocarditis-like phenotype has not been extensively explored. All of our FLNC-positive cases had a myocarditic picture, according to the European Society of Cardiology criteria when taking into account the clinical presentation, ECG, myocardial cytolysis markers, echocardiography, and tissue characterization on CMR.^4^ Consistent with previous associations between severe myocarditis and cardiomyopathy variants, all of our cases presented with significant LV dysfunction, ventricular arrhythmias, or sudden death; Patients 1–3 had a high burden of ventricular ectopy/non-sustained VT or VT; Patients 1–3 had relevant family history prior to the presentation; Patients 3 and 4 had severe LV systolic dysfunction and RV involvement, and Patients 4–6 had lymphocytic infiltration on histological examination of the heart; Patients 5 and 6 suffered sudden cardiac arrest. Cascade testing was useful in Cases 1, 4, and 6 for identifying other affected family members, leading to appropriate surveillance and preventative interventions. It is also interesting that all patients with a clinical/imaging-based diagnosis of myocarditis and ventricular arrhythmias exhibited a variable degree of QRS fragmentation in the inferior/lateral lead, which correlated with the presence of scarring on CMR. Studies have shown that QRS fragmentation is associated with ongoing inflammation and with a poorer outcome in terms of ventricular function and occurrence of arrhythmias in patients with acute myocarditis.^5^

It is increasingly recognized that patients with pathogenic variants known to be associated with dilated and ACM may present with acute or chronic myocarditis.^6–8^ Studies report an increased prevalence of pathogenic DCM or ACM variants between 8 and 31% of myocarditis cases, depending on the population studied, with the most commonly identified genes being desmoplakin, titin, FLNC, and RNA binding motif protein 20.^7^ Brown *et al.*^9^ identified likely pathogenic and pathogenic variants (MYBPC3, TTN, TNNT2, and SCN5A) in five of eight children hospitalized for acute heart failure caused by acute myocarditis, and in another cohort of 42 paediatric patients with myocarditis, at least one likely pathogenic/pathogenic variant (BAG3, DSP, LMNA, MYH7, TNNI3, TNNT2, and TTN) was identified in 9 out of 42 patients (22%).^10^ Myocardial inflammation is not uncommon in genetic ACM; inflammatory infiltrates are found in ∼80% of arrhythmogenic right ventricular cardiomyopathy (ARVC) patients using post-mortem histological analysis.^11^ A myocarditis-like phenotype with ‘hot phases’ is well characterized in desmoplakin cardiomyopathy exhibiting episodes of myocardial injury akin to myocarditis, preserved ventricular function, and subepicardial fibrosis in carriers of DSP variants.^12^ In a cohort of 21 cases of recurrent myocarditis, it was found that approximately one-third of patients had a previously unknown pathogenic or likely pathogenic mutation, mostly affecting the DSP gene.^13^ Additionally, Piriou *et al.*^14^ found that in patients with acute myocarditis and a family history of cardiomyopathy or sudden cardiac death, genetic testing revealed unknown or misdiagnosed arrhythmogenic variant carriers with left-dominant phenotypes that may evade the ARVC Task Force Criteria. An inflammatory insult may uncover increased genetic susceptibility to develop significant LV dysfunction or an arrhythmogenic phenotype in cases of acute myocarditis.^15^

It is also possible that an association between pathogenic or likely pathogenic variants and the development of severe myocarditis exists. Myocarditis patients with DCM variants tend to have a worse outcome with greater mortality compared with those with no variants.^16,17^ In another study, 4 of 12 patients (25%) with severe myocarditis carried a pathogenic or likely pathogenic variant, and FLNC gene was identified in 1 of those cases (8%).^17^ The diagnostic yield of genetic testing may be higher among patients presenting with myocarditis and sustained ventricular arrhythmias, right ventricular abnormalities, family history of cardiomyopathies, or sudden cardiac death.^4,5,18^

As well as clinical and imaging data supportive of myocarditis (for Cases 1–3), this case series is the first to provide histologic evidence of myocardial lymphocytic infiltration (for Cases 4–6) in FLNC mutation carriers, strengthening the hypothesis that genetic defects in cardiomyocyte structural proteins could confer increased vulnerability to myocardial inflammation triggered by a causative agent, such as viral infection (i.e. a ‘two-hit’ hypothesis). In conclusion, genetic testing for FLNC mutations, as well as appropriate family screening, may be considered in cases presenting with a picture of acute myocarditis accompanied by severe LV systolic dysfunction, biventricular failure, significant ventricular arrhythmias, RV involvement, or when there is family history of cardiomyopathy or sudden cardiac death. Genetic testing may also be considered in patients presenting with acute myocarditis and LGE distribution in a crescentic or ring-like pattern, which is often well defined and delineated from the surrounding myocardium as opposed to the often ill-defined and patchy distribution seen in viral myocarditis.

## Data Availability

The data underlying this article will be shared upon reasonable request to the corresponding author.

## Lead author biography

Apostolos Vrettos is a cardiology specialty registrar, sub-specializing in advanced heart failure and cardiac imaging at St Bartholomew’s Hospital, in London, UK. He graduated from the University of Athens Medical School in Greece, in 2012. He has completed a Master’s degree in Clinical Research and a Postgraduate Certificate in Medical Education and has always tried to be academically productive while clinically focused. He is an echocardiography passionate and biostatistics enthusiast, who enjoys teaching and mentoring junior colleagues, as well as exploring how advances in imaging techniques could improve patients’ outcomes.

## Notes

**Consent:** The authors confirm that written consent for submission and publication of this case report, including images and associated text, has been obtained from the patients or relatives (next of kin) in line with COPE guidance.

**Funding:** None declared.
